# Men’s social representations of the body across age groups

**DOI:** 10.1186/s41155-026-00386-1

**Published:** 2026-03-18

**Authors:** Ariane Franco Lopes Da Silva

**Affiliations:** 1 Faculty of Education and Psychology, Centre of Studies for Human Development (CEDH), Universidade Católica Portuguesa, Rua Diogo Botelho, 1327 – 4169-005, Porto, Portugal; 2 Ultreia Association, Rua do Campo Alegre, N1517 LJ24 C82, 4150-182, Porto, Portugal

**Keywords:** Social representations, Body image, Health, Men, Age

## Abstract

**Background:**

This study looks at how men of different ages represent their bodies through verbal and visual modalities and explores their aspirations for their bodies in the future. Drawing on the perspective of social representations, it highlights how culture, tradition, and personal experience shape perceptions of the body. Understanding these representations is important, as they influence how people think about and relate to their bodies.

**Objective:**

The study aims to map out everyday beliefs and social representations about the body produced by young and middle-aged men and observe their connections with health and well-being. The study also examines how men anticipate bodily changes over the life course, and which physical attributes are considered most ideal and valued.

**Methods:**

Using a mixed-method approach, the study explores bodily representations through word associations, and drawing techniques. A total of 112 men from Brazil aged 18 to 50 participated in this study. Group 1 consists of 68 men aged 18 to 25 and Group 2 consists of 44 men aged 26 to 50. The software Evoc (2000) was used to analyse data from the word association, while content and image analysis techniques were applied to examine textual and visual data from the image-based tasks.

**Results:**

The results show patterns in body-representations as well as unique and diverse perspectives on body, well-being, and health. They also highlight both adherence to norms and conventions and ongoing shifts in traditional views about the male body.

**Conclusion:**

Different age groups integrate cultural norms and personal life experiences in distinct ways. The study contributes to the literature on age-related differences in men’s body-representations and explores their possible implications for health related behaviour, and body maintenance protocols.

## Introduction

The body plays a crucial role in the study of the ways individuals see themselves and relate to others. It intersects with several disciplines, including social psychology, sociology, health, and non-verbal communication. Across these fields, the body has been examined as an important element in understanding how individuals present themselves in public and how they control their bodies in social interactions (Goffman, 1959). It has also been considered as a medium of non-verbal communication, where gestures, clothing, and physical appearance express emotions and attitudes (Argyle, 1994). Additionally, bodily expressions and appearance may signal social identities and group affiliations (Hall, 2011; Howard, 2011), with particular hair styles, body ornaments, and even the desire to have a specific body figure serving as symbols of both social and personal identities (Mair, 2018; Franco, 2015; Immergut, 2010; Silva & Cohen, 2025). Age, and the experience of aging, constitute one of these identities that bodily features often manifest, as has been investigated within the context of health. The work of Herzlich (2018), for instance, focuses on the notions of health as formed by a number of meanings such as activity, functionality, and autonomy, and demonstrates how valuable they are to older individuals because they sustain their ability to maintain social connections and roles within society.

While research of the body, appearance, and aging has traditionally focused on women, a new line of enquiry has emerged that examined the male body across the life course. This growing area of research reflects the increasing visibility of the male body in popular culture, which has subjected men to intensified scrutiny and pressure to conform to norms of appearance resulting in body dissatisfaction (Grogan, 2008). Additionally, other studies also point to a growing surge in men’s preoccupation with their appearance and its effects on physical and mental health (Rumsey & Harcourt, 2014). This preoccupation is evident in the pursuit of both thinness and muscularity, that represent societal male archetypes (Cacioli & Mussap, 2014; Cunningham et al., 2020; Eufrásio & Nóbrega, 2017; Girard et al., 2018; Tylka, 2011), and decisions about health-related behaviour, and body maintenance protocols (Hooker et al., 2012; Saltonstall, 1993). Other studies also looked at engagement with image-focused social media that promotes health and active life styles yielding both positive and adverse impacts on body image, physical and mental health (Hogue & Mills, 2019; Prichard et al., 2020; Raggatt et al., 2018; Rounsefell et al., 2020; Tiggemann & Zaccardo, 2015). Usage of image-focused social media has also been related to muscularity dissatisfaction among men (Griffiths et al., 2018).

Another body of knowledge focused on how men redefine their identities when they move into a different life stage. It observes complex negotiations between changes and continuity, often reflected in strategies and systems of check and balances, as well as in the maintenance of certain traditional masculine ideals (Gough, 2006; Davidson & Meadows, 2010). In line with these findings, research indicates that while younger men generally aspire to a muscular physique, with well-developed chest, arms, and shoulders, and report a large discrepancy between actual muscularity and body ideals (Pope et al., 2000), older men tend to display smaller discrepancies between current and ideal body shape, and express lower levels of body dissatisfaction (Barker & Gringart, 2012). Consistent with this pattern, studies indicate that older men become less involved in physical fitness and traditional masculine traits, more interested in health and enjoyment (Hardy & Grogan, 2009; Hooker et al., 2012), and more inclined to perceive the aging body as functional and intertwined with mental, spiritual, and health-oriented practices (Silveira et al., 2021).

Perceptions of growing old and the display of traditional male physiques have also been examined with research showing that experiences of masculinity and muscularity can differ across life stages, lifestyles, and personal experiences. Studies have highlighted that muscularity is often the product of bodybuilding regimes that are closely tied to hegemonic masculinity (Edwards et al., 2017). Masculinity is a socially constructed, relational, and hierarchical phenomenon, encompassing a set of characteristics, behaviour, and values typically associated with being a man, such as toughness, competitiveness, and physical strength (Connell, 1995). Hegemonic masculinity refers to the dominance of these attributes over other forms of gender expression, stablishing them as ideal ones (Connell, 1995). Thus, the pursue of highly muscular body and physical strength become means of embodying masculine identity, and by doing so increases one’s masculine capital (Edwards et al., 2017). However, Connell also recognizes that bodies are in a constant dialogue with the social world, and anchored in historical contexts. By highlighting this dynamic interplay, Connell (1995) emphasizes that individuals engage in varying degrees in reproducing dominant forms of masculinity, what allows the emergence of non-hegemonic forms of masculinities. This may help explain the diverse aspirations reported by older men regarding their bodies, shape, appearance, and health practices (Gough, 2006; Davidson & Meadows, 2010; Pope et al., 2000; Barker & Gringart, 2012; Hardy & Grogan, 2009; Hooker et al., 2012; Silveira et al., 2021).

### Social representations and the study of the body

Social representations are common-sense knowledge about a topic produced through the exchanges of everyday conversations, contextual characteristics, and personal experiences (Moscovici, 1961). Beyond guiding behaviour, and helping explain adherence to certain practices, social representations also orient the formation of assumptions, judgements, and expectations about the subject of representation (Moscovici, 1961). Social representations are produced by the processes of anchoring, which refers to the association of a new idea with a familiar one, and objectivation, which refers to the attribution of a form to ideas and beliefs, maintaining their correspondence to an external reality (Moliner, 2020).

The structural approach to this theory (Abric, 1994) states that social representations consist of a set of ideas about a social theme and have elements that are central and others that are peripheral. The central ideas are both conventional and descriptive and are more resistant to change. The peripheral ones, on the other hand, are contextual and related to individuals’ lived experiences, and more prone to changes. Methodological tools that use word associations are known to aid the identification of anchoring processes, by facilitating spontaneous expression of views about a topic, and their connections with traditions, and culture (Abric, 1994). That is, they help the understanding of how individuals perceive their changing bodies in relation to what is already known about it. Other methodological tools use image production and interpretation techniques to assist in the identification of objectivation processes, because they make ideas more visually accessible (Galli et al., 2017; Moliner, 2020; Nicholson & Awad, 2024), and translate bodies’ tangible and physical dimensions (Silva et al., 2024). In this sense, studying social representations of the body using a mixed-method approach provide a more nuanced insight into how common-sense knowledge is constructed and shared, and their associations with health across the life course.

### The social representation of the body: between the personal and the social

Some studies emphasize that identity is a position within a culture’s symbolic field, providing a sense of stability (Duveen, 2001). While individuals tend to adopt the characteristics, values, and practices of the group they are preparing to join, the group pressures them to clearly express that identity. However, Duveen also observed that identity depends on social representations, shaped by the continual dialogue between the individual and society, a dynamic process that recognizes individuals as active participants in the formation of representations.

From a complimentary perspective, Breakwell (2001) examines the different types of social representations and their relationship to personal ones. Drawing on Moscovici’s (1961) work, Breakwell highlights the varying degrees of individual freedom in constructing social representation. This framework identifies three types of representations: hegemonic, emancipated and polemical. The hegemonic representations are shared by all members of a group, leaving little room for individual variation, while polemical representations arise in the contexts of intergroup conflicts, and society as a whole does not share them. Emancipated representations, on the other hand, emerge within sub-groups that create their own version of that representation. Building on this framework, Breakwell (2001) discusses that personal representation emerge when emancipated and polemical representations prevail, promoting innovation and change. For the author, this process is likely to happen when people encounter social representations that might threaten important aspects of their identity. As a consequence, individuals may slightly change or adjust elements of these representations, while maintaining others untouched, what attest for the variety among individuals despite the existence of elements of agreement and consensus (Breakwell, 2001).

Drawing on these studies and connecting them with the issue of the social representation of the body across the life course, as men grow older, they may encounter social representations about the body that are more hegemonic and others that are emancipated or polemical. As a consequence, they may interpret these representations and while endorsing certain bodily habits and norms of conduct, and valuing certain body features known to define the members of the age group they are entering, assimilate others less hegemonic representations. This negotiation gives rise to a variety of ways individuals express themselves, engage in health related practices, and perceive themselves as active members of society.

### About the study

In light of the increased scrutiny of the male body and the growing pressure to conform to social norms, the present research aims to address this gab by examining how men of different ages represent their bodies across the life course. This study aims to map everyday beliefs and social representations about the body among young and middle-aged men in Brazil. It also examines how men anticipate bodily changes over time, by analysing body representations in the present and future, and which physical attributes they consider most ideal and valued. The goal is to identify shared knowledge about the characteristics, values, and practices associated with the age group they are expected to join later in life, as well as the extent to which individuals construct their own versions of body representations. The study poses the following questions: (1) How do men of different ages represent their bodies?; (2) How do they predict body changes across the life course?; (3) What body features are considered most ideal and valued? The study can contribute to the literature on age-related differences in men’s representations of the body, as well as their potential effects on physical and psychological health.

The theory of social representations provides the theoretical and methodological foundation for this study, highlighting the significance of common-sense knowledge about certain topics and the interplay between social realms and individuals’ lived experiences in shaping behaviour (Moscovici, 1961). Certain methodological tools that use verbal language are known to facilitate the expression of common-sense knowledge on various topics, support the analysis of the structure and content of social representations, and aid in identifying anchoring processes. Image production and interpretation techniques are also employed in research on social representations, aiding in the identification of objectification processes (Galli et al., 2017; Moliner, 2020; Nicholson & Awad, 2024; Silva et al., 2024).

For Moliner (2020), image production and interpretation require the mobilization of cultural knowledge about the object, and a shared understanding of the coding system that guarantees some kind of correspondence between the image and the object. Because images can translate shared views on diverse topics they are thought to be closely associated to social representations. This aligns with the idea that, because bodies may project both personal and shared characteristics (Jodelet, 2006), including those valued and meaningful within an age group, the perspective of social representations helps in understanding the intricate relationship between collective and individual perceptions of the body across the life course.

## Methods

This study is part of a broader research project, “Representations of the body and health: expression through images and words,” which investigates how the body contributes to the expression and formation of social representations among men and women of different ages. Using a mixed-method approach that integrates verbal and visual modalities, this project also supported several related studies. These include investigations into undergraduates’ beliefs about students’ identity and the university experience (Silva et al., 2020), and representations of women in the illustrated press and their role in non-formal education in the first half of the 20th century (Silva et al., 2021). More recently, comparative studies explored how women of different ages and countries perceive their bodies, highlighting both shared and divergent perspectives regarding body image, aging, and health (Silva et al., 2024; Silva & Cohen, 2025). Building on these prior studies and in light of the increased scrutiny of the male body and the growing pressure to conform to social norms, the present research seeks to address this gap by examining how men of different ages represent their bodies across the life course.

It consists of a questionnaire with word association, and drawing production techniques, that intends to facilitate an in-depth analysis of representations. The mixed-method approach also facilitates the identification of anchoring and objectivation processes and the visualization of diverse perspectives of social representations ((Flick, 2023; Martikainen & Hakoköngäs, 2023; Silva et al., 2024; Silva & Cohen, 2025).

### Participants

A total of 112 male subjects participated in this study. Group 1 (G1) consists of 68 men aged 18 to 25 (Mean - M = 20, Standard Deviation - SD = 1.92) and Group 2 (G2) consists of 44 men aged 26 to 50 (Mean - M = 34, Standard Deviation - SD = 6.9). The participants are originally from the southern region of Brazil. A convenience sample was recruited by approaching individuals in a variety of settings, including universities, workplaces, and retail environments. They were informed about the study’s aim, the voluntary nature of participation, the right to cancel participation at any time, and the anonymity of their data. Those who agreed to participate provided a written informed consent. Then, the participant received a printed, self-administered questionnaire, which was completed under the supervision of masters students.

According to information provided on income, the participants have a middle-class background, which corresponds to the average living standards of the region (Instituto Brasileiro de Geografia e Estatística, IBGE, 2022). In G1, 95.58% of participants are single, compared to only 34.09% in G2. As far as education is concerned, 95.58% of participants in G1 are enrolled in higher education, and 47.7% in G2 are either graduated or enrolled in master’s degrees. Almost half of participants in G1 are working part-time (48.52%), and 84.09% in G2 are working full time.

### Materials and procedures

The research instrument is divided into two main sections. In the first section, the participants were asked to write the first five words that came to mind after the following prompts: ‘body in the present’, and ‘body in the future’. The objective is to identify the most relevant ideas about the body as it is perceived in the present and projected to be in the future. The data were analysed by the software Evoc - *Ensemble de programmes permettant l’analyse des évocations* (2000) based on the method designed by Vergès (1992). A table was produced showing the combination of the frequency of evoked words with the mean rank of evocations of each prompt. The table provides an overview of the central words (high frequency and low order of evocation), and those at the peripheries (high frequency and high order of evocation, and low frequency and low order of evocation). The central words are thought to describe the representation, expressing ideas anchored in memories and social conventions. The words in the peripheral zones are thought to convey ideas more associated with individuals’ contexts and life situations. They may reinforce and complement the central core, or express different views, indicating possible changes under way to the representation. Each table also provides a comparative analysis between the two groups of participants.

In the second section of the study, the participants were asked to produce two drawings, according to the themes: ‘*The body I have’* and ‘*The body I would like to have*’. The instrument intends to examine how individuals translate their thoughts and beliefs about the appearance of the ideal body into a visual form. The aim of this technique is to observe the differences between the images of the actual and the desired body (Silva & Cohen, 2025). The task of making a drawing captures processes of selecting the items that correspond with what is known about the object being represented (Silva et al., 2020). It is particularly useful in studies aiming to examine objectivation processes in social representations, and the simultaneous representations of diverse ideas about a specific topic (Martikainen & Hakoköngäs, 2023; Silva & Cohen, 2025). Additionally, drawings provide a source of data on body ornaments, and physical traits because of their direct correspondence with the object being represented.

An initial floating analysis of the drawings was carried out, using the principles of the content analysis (Bardin, 1977). It produced an inventory of the prevailing physical features of the bodies. A second round of analysis allowed the observation of patterns in the representations, allowing for the formation of categories and sub-categories. The body features were then grouped into four main categories: weight, shape, facial hair, and body ornament. Next, each participant’s two drawings were compared to observe changes in the ideal body (drawing b.) in relation to the actual one (drawing a.). Thus, this procedure focused on identifying which body features were most altered in drawing b. Finally, the results of the two age groups were compared to determine whether they differed in how they represented the desired body.

The coding procedure used to classify the drawings according to weight and shape was supported by the studies of Cunningham et al. (2020), Eufrásio and Nóbrega (2017), and Griffith et al. (2018), which identify these features as relevant components of male body standards widely promoted in the media. The coding process to classify the drawings in terms of ornamentation and grooming, attributes that may shape perceptions of male social status, hygiene, and health, was based on the studies of Dixson and Vasey (2012), Dixson and Brooks (2013), Immergut (2010), and Ory (2011). Finally, the coding process regarding body modification and ornament such as tattooing, which can symbolize religious beliefs, group affiliation, social identities, and personality traits, was supported by the studies of DeMello (2007), Orbach (2009), and Wohlrab et al. (2007).

### Ethics approval and consent to participate

All ethical guidelines were followed and the necessary approvals for the research were obtained from the Human Research Ethics Committee of Universidade Federal da Fronteira Sul (CAAE number: 91848218.3.0000.5564; CEP number: 2.752.334).

## Results

### Free association

In the first section, the participants were asked to write down five words that came to mind after the following prompts: ‘body in the present’, and ‘body in the future’. The data were analysed using the software Evoc - *Ensemble de programmes permettant l’analyse des évocations* (2000). Table 1 shows the combination of the frequency and mean rank of evocations for each prompt across both groups (Group 1 - G1, and Group 2 - G2). The mean rank of appearance was selected to be 2.5, with lower mean ranks indicating words that were more readily evoked. The words that combine low rank of appearance with high frequency level are considered central to the description of representations (Abric, 1994). The words evoked by more than 10% of participants were considered as forming a pattern, reflecting the structure and content of the representation.

Table 1 shows that being healthy (“health/healthy”) emerged as the most frequent (G1, f = 19–27.94%; G2, f = 12–27.27%) and readily evoked concept, as evidenced by the low rank in the order of evocation (below 2.5 - G1 = 2.316; G2 = 1.917). For both age groups, health was a dominant theme in the description of the current body. The focus that participants in G2 place on health aligns with studies showing that men become less involved in physical fitness and traditional masculine traits, and more interested in health and enjoyment as they get older (Hardy & Grogan, 2009; Hooker et al., 2012; Barker & Gringart, 2012). Contrary to studies that point to the fact that young men aspire to a muscular body shape that represent societal male archetypes (Cacioli & Mussap, 2014; Cunningham et al., 2020; Tylka, 2011), participants in G1 did not mention muscle mass or body shape to describe their current bodies. But they mentioned strength, which may convey power, efficiency, activity, and energy, and be linked to activity levels than to muscularity alone. That was also the case in G2, a result that is also consistent with studies that point to traditional masculine traits being less valued among older men. Conversely, weight (thin/slim) and heigh (tall) were the physical characteristics chosen to describe the current body in G1. Possibly, young participants’ perspectives on the body shifted to include these physical traits, being “thin/slim” particularly descriptive of this representation because it is closely related to the central core of the representation (low rank of evocation).

**Table 1:** *Free association: ‘Body in the present’ and “Body in the future”- Software Evoc - Ensemble de programmes permettant l’analyse des évocations (2000)*, *frequencies and mean rank of appearance (f*,* %*,* and Rank) - (G1–68 participants; G2–44 participants)*

G1				G2			
Body in the present							
Words	f	%	Rank	Words	f	%	Rank
Health/healthy*	19	27.94	2.316	Health/healthy*	12	27.27	1.917
Handsome	9	13.23	3.111	Active/exercise	12	27.27	3.833
Thin/slim	8	11.76	1.250	Strong	8	18.18	2.250
Tall	8	11.76	2.625	Handsome	7	15.90	2.857
Happy	8	11.76	3.375	Thin/slim	6	13.63	2.000
Fat/heavy	8	11.76	3.375				
Strong	8	11.76	3.500				
**Body in the future**							
Words	f	%	Rank	Words	f	%	Rank
Strong*	8	18.18	2.000	Health*	21	47.72	2.476
Health	20	45.45	2.700	Strong	8	18.18	3.000
Handsome	10	22.72	3.500	Care	8	18.18	3.250
Improved	7	15.90	2.714	Old	7	15.90	1.429
Old	7	15.90	3.250	Thin/slim	7	15.90	2.286

* Central words (combine lower mean rank of appearance with higher frequency of evocation)

For the prompt ‘body in the future’ (Table 1), the groups showed slightly different responses. G1 emphasized physical power, with the word “strong” appearing as the central core of the representation, complemented by “health” and “handsome” in the peripheries. An analysis of the results reveals that being strong and muscular are hegemonic representations of the male physique (Connel, 1995), and that was evidenced in G1. But being handsome depends on more subjective evaluations, based on social and cultural standards, and encompasses a broader range of traits, including facial characteristics, grooming behaviour, clothing, and so on. Being handsome is an element that forms a representation of the present body that is carried to the future body by participants in G1.

Conversely, the findings from G2 for the prompt ‘body in the future’ (Table 1) show that the word “health” (f = 21) was the most frequent and promptly evoked, followed by “strong” (f = 8) and “care” (f = 8). This result shows the prevalence of health concerns among older men and less variations in their perspectives about the older body than among younger participants. This result is consistent with the studies about the importance of health, functionality, autonomy, and activity for older individuals (Herzlich, 2018). The words “old” (f = 7) and “care” (f = 8) at the peripheries reinforce this centrality by emphasising awareness of potential health challenges in the future and their impact on autonomy. Being handsome was not present in this group, an absence that may indicate less expectations or value to this particular characteristics in older age.

The analysis also looked at the words mentioned just once or with low frequency of appearance. They often conveyed different meanings and could not show any patterns or form categories. This measure is designed to identify variations in representations and understand their structure and content. Items with low frequency can reflect the extent to which ideas about the male body are dispersed withing the groups, indicating lower consensus, and greater diversity of perspectives. This approach is supported by the investigations of Duveen (2001) and Breakwell (2001) who showed that social representations often exhibit both consensus and a range of variations within the same group due to the dynamic dialogue between personal experiences and traditions in their formation of representations. Thus, this analysis could reveal a range of variations within the same representation.

In G1, the prompt ‘body in the present’ elicited a high number of words with different meanings (59.36%), such as “food” (f = 6, 8.82%), “tired” (f = 6, 8.82%), “exercise” (f = 6, 8.82%), “love” (f = 5, 7.35%), “weak” (f = 4, 5.88%) and “satisfied” (f = 4, 5.88%). G2 presented a slightly lower number of these words (50.22%) compared to G1, such as “well-being” (f = 3, 6.81%), “mind” (f = 2, 4.54%), and “friends” (f = 1, 2.27%). This result supports the data in Table 1 and indicates that G2 presents less variations in perspectives about the current body than G1. The number of words with low frequencies of evocation or mentioned just once for the prompt ‘body in the future’ was higher than that for the previous prompt in both groups (G1 = 68.54%; G2 = 56.22%). Participants in G1 displayed even more varied perspectives on the older body than those from G2, including “married” (f = 2, 2.94%), “gym” (f = 1, 1.47%), “wrinkles” (f = 1, 1.47%), “decline” (f = 1, 1.47%), and “joy” (f = 1.47%). In G2, examples consist of “pain” (f = 3, 6.81%), “limit” (f = 2, 4.54), “free” (f = 1, 2.27%), and “spirituality” (f = 1, 2.27%).

This outcome may be attributed to two factors. More subtle changes to the body would be evoked if subjects considered a distant future. Thus, younger men would have a longer timeframe to reflect on when representing their aging body, resulting in a greater variety of perspectives than older men. Alternatively, the findings could stem from the aging body not being a topic of concern yet for younger men. As a result, there is less discussions, consensus, or shared ideas on the subject, giving room for the expression of more subjective and varied views. Further research is needed to explore the effects of varying time frames on individuals’ anticipatory representations of their bodies. For older men, aging may be a topic of ongoing discussions, and with time, conversations create agreements and shared views.

### Drawing the body

In the second phase of the study subjects were asked to produce two drawings side by side following the themes: (a) ‘*The body I have’*, and (b) ‘*The body I would like to have’*. Initially, a random analysis of the drawings was carried out with the aim to produce an inventory of the main physical characteristics, observe patterns in representations and form categories. Once the most common body features were identified, they were classified in four categories: weight, shape, facial hair, and body ornament. These prevailing physical features align with those often examined by scholars. Slender and muscular bodies (categories weight and shape) are key aspects of male beauty standards (Cunningham et al., 2020; Eufrásio & Nóbrega, 2017; Griffiths et al., 2018), and have been widely promoted in the media. The pressure to meet these standards has been linked to rising body dissatisfaction among young men (Galioto & Crowther, 2013). When it comes to body ornamentation and grooming (categories facial hair and body ornaments), hair care and beard styles play a significant role in shaping perceptions of male social status and aggressiveness (Dixson & Vasey, 2012; Immergut, 2010), masculinity (Dixson & Brooks, 2013), and conceptions of hygiene and health (Ory, 2011). Regarding practices of body modification and ornament such as tattooing, they often symbolize religious beliefs, group affiliation, social identities, and personality traits (DeMello, 2007; Orbach, 2009; Wohlrab et al., 2007).

The category ‘weight’ refers to rounder or slimmer figures. This category has three main sub-categories: ‘light’, ‘heavy’, and ‘unclear’. Drawings with slimmer body parts were classified as ‘light’, and those with larger and rounder stomach were classified as ‘heavy’. Drawings with no clear references to weight changes were classified as ‘unclear’. An example of drawings in this category includes figures with such minor alterations to their waistline that no conclusions could be drawn about an intention to depict weight alterations. The category ‘shape’ refers to muscle mass/tone. This category has three sub-categories: ‘low muscular definition’, ‘high muscular definition’, and ‘unclear’. Drawings with slender figures and few muscular definitions of the waist, chest, arms, and legs were classified as ‘low muscular definition’, and toned figures with rounder and more muscle mass in these body parts were classified as ‘high muscular definition’. Drawings with no clear references to muscle mass were classified as ‘unclear’. An example of drawings in this category includes figures dressed with long-sleeved shirts and loose-fitting trousers that concealed their muscle tone.

The category ‘beard’ has two sub-categories: ‘with beard’ and ‘without beard’. In this case, categorizing the drawing into either sub-category was straightforward because no other factors obscured the depictions of facial hair. The category ‘hair’ has two sub-categories referring to hair style, and length: ‘short/less hair’, ‘long/more hair’. In this case too, categorizing the drawing into either sub-category was simple because no other factors compromised this depiction. The category ‘body adornment’ refers to tattooing. This category has two sub-categories: ‘with tattoo” and ‘without tattoo”. The categorization was based on whether the figures had tattoos, what directly assigned each drawing to one sub-category or the other.

The data were analysed to observe differences between drawing (a) and drawing b., focusing on contrasts that might indicate dissatisfaction with the body. To explore this further, a more detailed examination of drawing (b) was conducted using the categories and sub-categories. Additionally, a comparative analysis between groups was performed to examine age-related differences in self-representations.

**Fig. 1:**
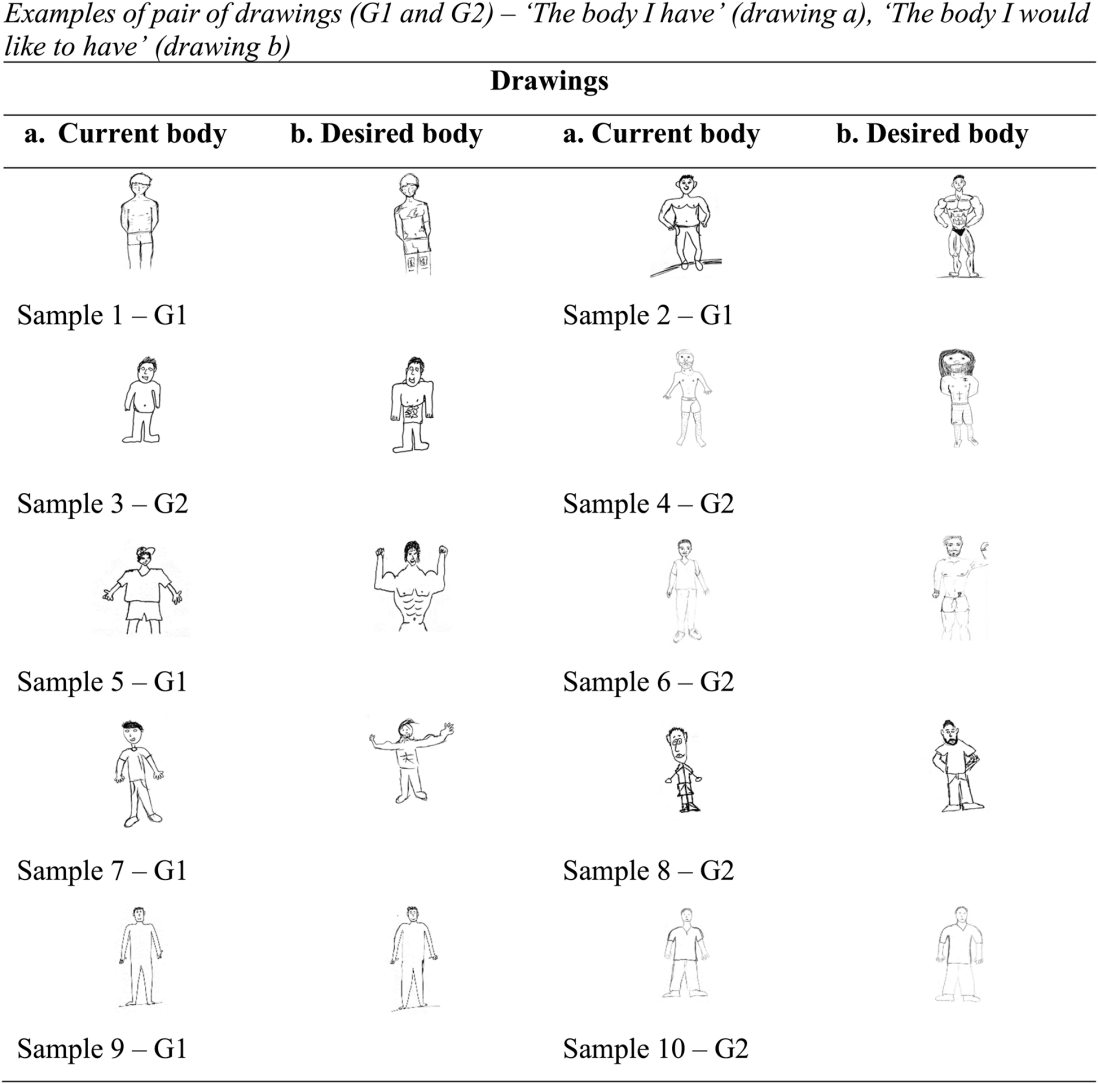
Examples of pairs of drawings

Pairs of drawings can be seen in Fig. 1, with the current body (drawing a) next to the ideal body (drawing b) of the same participant. Sample 1 (G1) illustrates differences in body shape exemplified by modifications in the chest and shoulder areas, from low muscular definition (a) to high muscular definition (b). There were also differences regarding body ornaments, with the addition of tattoos in the chest and legs (drawing b). Sample 2 (G1) illustrates differences in body weight and shape, from rounder stomach and low muscular definition (a), to slimmer waist and high muscular definition (b), exemplified by modifications added to waist, chest, arms, shoulders, and legs. Samples 3 (G1) illustrates differences between figures a. and b., highlighted by slender and more muscular arms, shoulders and stomach, and line drawings in the abdomen (b). Sample 4 (G2), show drawing b with longer and thicker hair, and higher muscular definition than drawing (a) and a tattoo in the chest. Samples 5 (G1), 7 (G1), and 8 (G2) show alterations to body shape, facial hair, and body ornament, characterized by more muscular mass in shoulders and arms, thicker beard and hair in all three drawings (b) Tattoos are more apparent in Samples 1 (G1), 4 (G2) and 8 (G2). Samples 9 (G1) and 10 (G2) show drawings (a) and (b) without changes.

Most subjects introduced at least one alteration to the second drawing (drawing b.) to depict a desired physique (G1 - *N* = 59, 86.76%; and G2 - *N* = 35, 79.54%), and G1 scored higher in this aspect. This may indicate a greater degree of body dissatisfaction or a stronger desire to improve and alter body shape in G1 compared to G2. This finding is consistent with studies that indicate that younger men generally aspire to a muscular physique, and report a large discrepancy between actual muscularity and body ideals (Pope et al., 2000). Fewer alterations to the second drawing (drawing b) by participants in G2 may indicate that older men are more accepting of bodily changes over the life course (Hardy & Grogan, 2009; Hooker et al., 2012; Silveira et al., 2021). This could also reflect a greater emphasis on mental health and staying active, consistent with studies by Hardy and Grogan (2009), and Hooker et al. (2012). Additionally, more participants in G2 preferred a slimmer body (45.71%) than those in G1 (38.9%). Slimmer bodies could be associated with being healthy.

Further analysis looked more closely at which body features were altered the most in drawing b. Table 2 shows that weight and muscle mass were frequently changed in both groups, showing a general preference for slimmer and muscular bodies. More participants in G1 preferred muscular bodies (59.32%) compared to G2 (45.71%). These results align with studies about the pursuit of thinness and muscularity as representative of male archetypes (Cacioli & Mussap, 2014; Cunningham et al., 2020; Eufrásio & Nóbrega, 2017; Girard et al., 2018; Tylka, 2011). Other research has paid particular attention to muscularity, investigating how engagement with image-focused social media relates to men’s dissatisfaction with their muscle tone (Griffiths et al., 2018). This may also help explain why more young participants, who are particularly targeted by social media platforms, wished to modify their bodies to increase muscle mass.

When comparing the current and the desired drawings in both groups, differences to the beard and hair were less common, evidenced by the higher frequency of drawings classified in the sub-category “no difference”. However, when alterations were made, participants preferred a thicker beard and a longer and thicker hair style. Participants in G2 scored higher than G1, pointing to a concern with hair loss in older age. Tattoos were also rarely used as elements that differentiate the current body from the desired one. Tattoos on the arms, shoulders, and lower legs were more frequent in G1 than in G2. As tattoos are often considered physically attractive and symbolise group membership (DeMello, 2007; Orbach, 2009; Maisey et al., 1999; Wohlrab et al., 2007) they may serve as a seductive devise, especially among younger adults, and express adherence to a particular group.

**Table 2:** *Drawing analysis – types of alterations to drawing b. (the ideal body) - categories and sub-categories – number and percentage (n and %) (items analysed - G1 = 59*,* and - G2 = 35)*

Categories	Sub-categories	Group 1, *n* (%)	Group 2, *n* (%)
Weight	Slimmer	23 (38.9%)	16 (45.7%)
	Heavier	8 (13.55%)	5 (14.28%)
	Unclear	28 (47.45%)	14 (40%)
Shape	Higher muscular definition	35 (59.32%)	16 (45.7%)
	Lower muscular definition	-	-
	Unclear	24 (40.67%)	19 (54.28%)
Beard	With beard/thicker beard	11 (18.64%)	5 (14.28%)
	Without beard/less beard	1 (1.69%)	1 (2.85%)
	No difference	47 (79.66%)	29 (82.85%)
Hair	With longer/thicker hair	8 (13.55%)	9 (25.7%)
	With shorter/less hair	2 (3.38%)	-
	No difference	49 (83.05%)	26 (74.28%)
Ornament	With tattoos/more tattoos	7 (11.86%)	1 (2.85%)
	Withought tattoos/less tattoos	-	-
	No difference	52 (88.13%)	34 (97.14%)

Studies have shown that particular hair styles, and body ornaments function as symbols of both social and personal identities (Mair, 2018; Franco, 2015; Immergut, 2010; Silva & Cohen, 2025), and that these features can often work as clues of age, and be markers for age group categorization. The findings indicate that hair appearance, and the desire to have more and thicker hair, reflect strategies used by older participants to disguise the signs of aging.

To refine the analysis further, a cross-examination of the most common categories was carried out to observe co-occurences of items (Silva et al., 2021, 2024). The intersection of the subcategory ‘higher muscular definition’ (G1 = 35, 59.32%; G2 = 16, 45.7%) with the subcategory ‘slimmer’ ‘(G1 = 23, 38.9%; G2 = 16, 45.7%) shows that G1 produced more drawings combining muscular and lean figures (G1 = 16, 45.71% ) than G2 (G2 = 4, 25%). The intersection of the subcategory ‘higher muscular definition’ with the subcategory ‘with beard/thicker beard’ shows that some participants combined these items, with those in G1 scoring higher than those in G2 (G1 = 10, 28.57%; G2 = 2, 12.5%).

A more detailed analysis of drawings b. classified under the sub-category ‘high muscular definition’ reveals variations to the position of the arms. Postures and gestures were not part of the classification process, because they were not sufficiently present in both drawings. However, changes to the position of the arms, and hands may add new elements to the depiction of the muscular physique and to the expression of the ideal male archetypes. Variations to the position of the arms include one or two arms stretched sideways in an open position, arms bent at the elbows with hands on the hips, arms bent with clenched fists, and hands at the back with elbows turned outwards (see samples 2, 4, 6, 7, and 8, in Fig. 1). In G1, 10 out of 35 drawings (28.57%) featured open arms, compared to 9 out of 16 drawings (56.25%) in G2. The open position of the arms may be intended to expose the body and clearly display the tattoos and muscle definitions. Although muscular definition was less common among the older participants, they depicted a combination of muscle tone and arms positions more frequently than younger participants.

## Discussion

The study observed what men of different ages think about their bodies as they are now and how they anticipate changes in the future. It identified hegemonic social representations, those widely shared across the groups. These shared ideas, views, images, and values provide members with a sense of stability, as they reinforce individuals’ positions within the group, as discussed by Duveen (2001). The representations were mainly anchored in the idea of health, strength, and activity, notions known to be especially important for older individuals because they support continued social participation and social connections (Herzlich, 2018). The results of the word association show that younger participants also share the view that health is important in older age.

It was also possible to identify a variety of traits associated with the body in both groups and across both conditions (present and future), resulting in different combinations. These traits may be shaped by unique life experiences that interact with more hegemonic representations, giving rise to new ones. These results can be interpreted through Breakwell’s (2001) examination of the different types of social representations and their relationship to personal representations. According to the author, personal representations emerge when different views produced by sub-groups prevail, facilitating innovation and change, despite still maintaining certain consensual views (Breakwell, 2001).

While health was a consensual theme, describing the representation of the body, anchored in the idea that a healthy body provides autonomy, maintains social relations and participation in society, other words conveyed more subjective ideas such as improved, happy, handsome, and care. Being attractive, illustrated for instance by the word ‘handsome’ in the word association task, may be considered an emancipated social representation, as it appeared in all cases, but less frequently. However, the absence of ‘being handsome’ in older participants descriptions of their future bodies suggests that this group of participants does not perceive a lack of attractiveness as a threat to important aspects of their identity.

On the other hand, ‘care’ was mentioned only by some older participants, indicating that they predict that care will be needed in the future. Thus, while still reflecting the hegemonic view represented by the word health, some participants in G2 incorporated care into the structure of their representation, possibly expanding its meaning to include some kind of management, maintenance, control, and guidance. In this sense, it gives a personal dimension to that representation. Finally, words illustrate shared as well as the unique views, that integrated form the social representation of the body. The analysis of words with low frequencies and those with single occurrences shows how the peripheries of representations are formed, and reveals potential transformations going on in the representation. Thus, the greater range of ideas in G1 compared to G2 may reflect ongoing shifts in traditional views of the male body in this group.

The drawing task allowed the observation of various physical qualities of the body as it enabled the expression of its physical aspects more than the word association task. It also provided data on objectivation processes, through depictions of physical features known to be valued in a group of individuals. In this sense, participants of both groups agreed on two body traits to wish for: slimmer and muscular. Representations of the current body were often critical, particularly regarding shape and weight, as evidenced by the alterations made in the desired body. However, G1 seemed more concerned with body monitoring regimes regarding muscle tone than G2, as reflected in the higher frequency of drawings classified in the sub-category higher muscle-definition. Participants in G2 seemed more preoccupied with weight than G1, because of the higher frequency of lean figures in their depictions of the desired body. The pressure to look slender (Grogan, 2008), the desire to keep an active life style (Barker & Gringart, 2012), and maintain good health together may explain the wish for a thinner figure. That aligns with studies showing that as men grow older, they become less involved in physical fitness and traditional masculine traits (Hardy & Grogan, 2009; Hooker et al., 2012).

Beards and hair complemented the desired muscular and lean figure in different ways, with thicker hair appearing as a desired characteristic in older men compared to younger ones. Thus, drawings visually illustrate subtle differences and preferred traits of body form, physical attributes and body ornaments, reflecting the co-existence of individual and collective ideas.

### Limitations and future directions

It was not possible to observe how far into the future participants imagined their bodies to be after the prompt ‘body in the future’. Different time spans could result in varied representations of the ageing body. Future research is needed to analyze more subtle differences in body representations in different time spans. Secondly, because this study did not obtain information about participants’ social media usage, it is not possible to examine its effect on different age groups. Further studies are needed to investigate the effect of social media and body practices in older men. Although some men over the age of 50 agreed to participate in the study, it was not possible to obtain a sufficient number to allow meaningful comparisons with the other age groups. Additional time and further studies are needed to reach a larger number of participants over 50. While the study observed variations in body representations across age groups, further longitudinal research is required to examine how these changes happen and to investigate potential differences within more narrowly defined age ranges. It was not possible in this study to examine how profession, work context, or working environment might influence body perceptions. Future research could more thoroughly investigate the impact of work context on body representations. The generalizability of the data could be a limitation. The findings may not extend to a broader group of men.

## Conclusion

This study expands our understanding of how men represent their bodies, highlighting differences across age groups. The mixed-method approach, with word association, and drawing production techniques, facilitated an in-depth analysis of the social representation of the body, the identification of anchoring and objectivation processes, and the visualization of patterns and unique representations. While certain body features represented how men would ideally like their bodies to be, younger participants displayed a wider range of beliefs about their bodies, and health, whereas older participants showed greater unity in their views. The age differences may be a result of the ways cultures, traditions, and individual’s lived realities interact in shaping social representations, highlighting both adherence to norms and conventions and ongoing shifts in traditional views of the male body, especially among younger participants. It is important to examine the elements that make up these representations, both shared and individual, traditional and new, because they are thought to guide attitudes and behaviour towards the body and health, and influence interpersonal relationships. The study contributes with the literature on age related differences on men’s body representations, and their potential effects on physical and psychological health, providing insights for community-based educational and health programs.

## Data Availability

The datasets used during the current study are available from the corresponding author on reasonable request.

## Abbreviations

DNA: Deoxyribonucleic acid
EVOC: Software - Ensemble de programmes permettant l’analyse des évocations (2000)
IBGE: Instituto Brasileiro de Geografia e Estatística
